# Understanding the Psychological Well-being of Patients With Locked-in Syndrome: A Scoping Review

**DOI:** 10.7759/cureus.34295

**Published:** 2023-01-27

**Authors:** Hiroshi Yoshiki, Nobuhisa Morimoto, Kevin Y Urayama

**Affiliations:** 1 Public Health, St. Luke's International University, Tokyo, JPN; 2 Nephrology, Tokyo Medical and Dental University, Tokyo, JPN; 3 Social Medicine, National Center for Child Health and Development, Tokyo, JPN

**Keywords:** resilience and well-being, end of life ethics, quality of life, locked in state, locked in syndrome

## Abstract

Locked-in syndrome (LiS) is a neurological disorder caused by lesions affecting the ventral pons and midbrain and is characterized by loss of physical function, but with perceived consciousness intact. Despite severely limited function, previous studies have shown the quality of life (QoL) in patients to be more positive than naturally assumed by caregivers and relatives. The present review aims to synthesize the broad scientific literature focused on the psychological well-being of LiS patients.

A scoping review was performed to synthesize the available evidence on the psychological well-being of LiS patients. Eligible studies included those that target individuals with LiS as the study population, evaluated psychological well-being, and explored the factors related to it. We extracted study population details, type of QoL methods, method of communication, and primary findings from the studies. We summarized the findings categorized into health-related QoL (HRQoL), global QoL, and other tools for assessing psychological status.

Across the 13 eligible studies, we observed that patients with LiS had reasonable or similar psychological well-being as the standard based on HRQoL and global QoL assessment. Caregivers and healthcare professionals seem to rate the psychological QoL of LiS patients lower than patients themselves. Studies showed evidence that the longer duration of LiS is a factor that positively affects QoL, and augmentative and alternative communication tools and recovery of speech production showed positive effects as well. Studies reported a range of 27% to 68% of patients experiencing thoughts of suicide and euthanasia.

The evidence shows that LiS patients had reasonable psychological well-being. There appear to be differences between patients’ assessed well-being and the negative perceptions by caregivers. Response shift and adaptation to disease by patients are considered potential reasons. A sufficient moratorium period and provision of information to support patients’ QoL and appropriate decision-making seems necessary.

## Introduction and background

Locked-in syndrome (LiS) is a rare neurological disorder caused by lesions affecting the ventral pons and midbrain. Injuries to the ventral pons, often due to stroke (ischemic and hemorrhage) are the most common causes of LiS. Additional conditions that can cause LiS include infection in certain portions of the brain, tumors, loss of the protective insulation (myelin) that surrounds nerve cells (myelinolysis), inflammation of the nerves (polymyositis), and certain disorders such as amyotrophic lateral sclerosis (ALS) [1]. Locked-in syndrome is characterized by patients having a limited motor function (except for vertical eye movement and blinking), but still having the intention or perceived consciousness with five senses and the ability of thought intact. As a result, independence and communication are severely impaired. It is differentiated from coma, consciousness disorder, and vegetative state, which manifest impaired consciousness, without awareness of the self and surroundings and with no voluntary motor movements [2].

The classical form of LiS is defined as quadriplegia and anarthria with the preservation of the ability to perform vertical eye movements, blinking, and maintaining a normal level of consciousness. The incomplete form is similar to the classical form, but with limited voluntary motor functions and movement. The total form is complete immobility and loss of function including eye movement, but with consciousness intact. In the classical and incomplete forms, consciousness is often evaluated by blink-response or eye movement-response to questions [3]. Reported mortality rates for LiS vary by study and etiology; there is a high risk of mortality in acute settings, but improved medical care approaches have improved long-term outcomes. Patients with medical stability of three years from onset showed a 10-year survival rate of 83% [4]. While the overall prevalence of LiS is largely unknown with variation by country, Kohnen et al. reported the prevalence of classic LiS in a Dutch nursing home setting to be 0.7 per 10,000 and suggested that this may be a low figure influenced by the Dutch provision of home care or end-of-life decisions (e.g., euthanasia, withholding or withdrawing medical interventions) [5]. Furthermore, this study brings to light the vitally important ethical issues and fundamental questions, such as euthanasia and end-of-life considerations while in LiS.

Contrary to the known limits in physical function, previous studies have shown that the quality of life (QoL) of LiS patients may not be as poor as initially perceived by caregivers [6]. However, most research has been based on small sample sizes due to the rarity of the condition, and studies have varied in the measures and approach used for QoL assessment as well as the etiology of LiS targeted for examination. In a recent systematic review on the prognosis and management of LiS patients, Halan et al. described that LiS patients had poor QoL, but noted this to be due to motor function disability which may be separate from depressive and psychiatric ailments of LiS [7]. Given the lack of cumulative knowledge on the psychological well-being of LiS patients, there is a need to summarize what is known based on current evidence from the scientific literature, including an understanding of the types of psychological and QoL instruments and measures used across LiS patients of varying etiology.

In this study, we performed a scoping review with the objective to understand the available evidence on the psychological well-being of LiS patients. We expect that this review will help identify the gaps in knowledge and inform areas for further research and development. This scoping review may serve as a precursor to a more targeted systematic review in the future and offer caregivers and relatives a more accurate perception of the LiS state.

## Review

Methods

A scoping review was performed to synthesize the available evidence from a broad perspective on the psychological well-being of LiS patients. We followed the Preferred Reporting Items for Systematic Reviews and Meta-Analyses extension for scoping review (PRISMA SCR) which guides the methodological process for scoping reviews. Considering the small sample size of LiS cases across the specific studies and the lack of established measures for evaluating the QoL of LiS, a scoping review was considered a reasonable approach to achieving study goals.

Protocol and Registration

We developed a protocol in line with the methodological framework by Arksey and O’Malley [8], which was later revised by Levac et al. [9]. 

Eligibility Criteria

The search considered original research articles that included human subjects of both qualitative and quantitative design and were written in English. Reviews, expert opinions, and policy documents were excluded. Articles with the following characteristics were included: 1) patients with LiS targeted as the study population, and 2) evaluation of the psychological well-being of patients with LiS and exploration of the elements influencing it such as LiS etiology.

Information Sources and Search Strategy

We searched the following databases (as of May 27, 2022): MEDLINE [PubMed], Cochrane Library and Embase related to the psychological well-being of LiS published within the last 30 years. Search terms (medical subject headings (MeSH) term) included (“Locked-in syndrome” OR “Locked-in state”) AND (“Quality of life” OR “well-being” OR “psychological”) in the title or abstract.

Study Selection

The references extracted from the databases were imported into an article screening tool Rayyan (Rayyan Systems, Cambridge, MA, USA) which can detect duplicated references. Two investigators (HY and NM) reviewed the titles and abstracts independently examining the references based on eligibility criteria. If a study appeared to meet the inclusion but there was doubt regarding the eligibility of the article, the full-text review was conducted for each article by both investigators. If a disagreement could not be resolved, a third reviewer (KU) was included.

Data Items

Data for extraction included publication details, study population details, type of QoL methods, and method of communication. Publication details included authors, year, country of study, and study design. Participants’ details included the number of participants, mean age, gender, etiology of LiS, the average time from onset, residential environment, and physical status. Communication methods included whether patients expressed intention by eye-blinking, use of augmentative and alternative communication (AAC) systems, face movement, residual verbal approaches, or a letter board. The type of person who interacted with patients to complete the QoL assessment in each study (investigator, caregiver or healthcare professional, etc.) was also noted.

Evaluation Approach

We divided the type of QoL and psychological well-being tool into three groups, including health-related QoL (HRQoL) designed for the general population, global QoL (subjective QoL) developed for intractable diseases, and other tools for assessing psychological status. In addition, we categorized the study's results into “reasonable results” for LiS patients (reasonable) and “negative results” for LiS patients (negative) in the case that each psychological well-being tool used in the study had the standard/reference score, or the study compared the psychological well-being status between LiS patients and control group. If each psychological well-being tool in the study showed better or similar outcomes in LiS patients compared to its standard/reference score or control group, we defined the result as “reasonable”. In contrast, when the study tool showed a worse result in the LiS group, we stated the result as “negative”. All validated psychological well-being tools used in the study are included in this review and their standard/ reference score are shown in Table 1 (this table doesn’t include the original questionnaire created specifically for the study). We evaluated the articles based on various aspects, such as the type of instruments used, patient characteristics, disease duration, country of origin, religion, and other exploratory factors.

**Table 1:** The instruments used to measure the quality of life and psychological status among the studies QoL: Quality of life, HRQoL: Health-related QoL, ALS: Amyotrophic lateral sclerosis

ID	QoL/ Psychological exam	Category	Adoption number in this review	Description	Standard or reference score
1	Anamnestic Comparative Self Assessment (ACSA)	Global QoL	4	The patient is asked to judge his or her global quality of life in relation to the worst (−5 on the Likert scale) and best (+5) experience in one’s own life	(≥0 indicates positive QoL)
2	Schedule for the Evaluation of Individual Quality of Life-Direct Weighting (SEIQoL-DW)	Global QoL	2	Determine the 5 most relevant fields of QoL, the share of each field for the subjective QoL, and the overall satisfaction with this field.	50 (range 0% to 100%; ≥50 indicates satisfactory QoL)
3	ALS Depression Inventory–12 (ADI-12) items	Depressiveness for ALS^1^ patient	2	Addresses the patient’s affective state. Response options are: fully agree (1) to fully disagree (4), adding up to scores 12-48	<28=normal
4	Schedule of Attitudes toward Hastened Death (SAHD)	End-of-life	1	Patients indicated a wish for hastened death that was assessed with the SAHD by providing binary options (correct/wrong) to respond to 20 statements	<10=not clinically significant wish
5	Motor Neuron Disease Coping Scale	Coping status	1	22 items that can be subsumed under 6 subscales of support, positive action, independence, avoidance, information seeking, and positive thinking.	>4=positive
6	SF-36/RAND-36* *scoring of pain and general health are different	HRQoL^2^	6	Consists of 8 items: physical functioning, role physical, bodily pain, general health, vitality, social functioning, role emotional, mental health	Reference score is different according to each region
7	Beck Depression Inventory (BDI-II)	Depressiveness	2	Response to 11 statements: (1) I do not feel sad. (2) I feel sad. (3) I am sad all the time and I can't snap out of it. (4) I am so sad or unhappy that I can't stand it.	0–9: indicates normal or minimal depression; 10–18: indicates mild depression; 19–29: indicates moderate depression; 30–63: indicates severe depression
8	Impact on Participation and Autonomy (IPA-E)	Autonomy	1	Score 0 (very good) to 4 (very poor) on five domains, 39 questions on autonomy indoors, family role, autonomy outdoors, social life and relationships, work and education	Reference mean is 1.48 in the social life domain; Iranian stroke population assessed 5 to 36 months after their stroke.
9	Euro QoL 5 Dimensions (EQ-5D)	HRQoL	1	Assesses health in five dimensions: mobility, self-care, usual activities, pain/discomfort, and anxiety/depression	A score under 0 is described as indicating a condition worse than death
10	WHO-5	HRQoL	1	Rating on 5 items: (1) I have felt cheerful, (2) I have felt calm, (3) I have felt active and vigorous, (4) I woke up feeling fresh and rested, (5) Daily life has been filled with things that interest me.	>60 means better (totally feel them more than half of the time)
11	McGill QoL (MQoL)	Global QoL	2	Rate 0-10 on 16 items. 1-4: physical, 5-8: psychological, 9-14: existential, 15-16: support area MQoL-EW=existential well-being, MQoL-Ps= psychological symptoms	No reference score
12	Hospital Anxiety and Depression (HADS)	Scale for depression and anxiety	1	Rate 0-4 on 7 items for each depression and anxiety	<7=normal for each item, 8-10=boarderline, >10=abnormal
13	Hamilton Anxiety Rating Scale (HAM-A)	Measure the severity of anxiety	1	Rate a scale of 0 (not present) to 4 (severe) on 14 items	14-17 indicates mild severity, 18–24 mild to moderate severity, and 25–30 moderate to severe
14	Tront Alexithymia Scale (TAS)	Alexithymia	2	A 20-item instrument that is one of the most commonly used measures of alexithymia	Equal to or less than 51=no alexithymia; scores of 52–60: possible alexithymia; equal to or greater than 61=alexithymia
15	State-Trait Anxiety Inventory (STAI-Y)	Anxiety	1	40 self-report items on a 4-point Likert scale. Measures two types of anxiety i.e., state anxiety and trait anxiety	The cut point of 39-40 has been suggested to detect clinically significant symptoms for the S-Anxiety scale

Results

**Figure 1:**
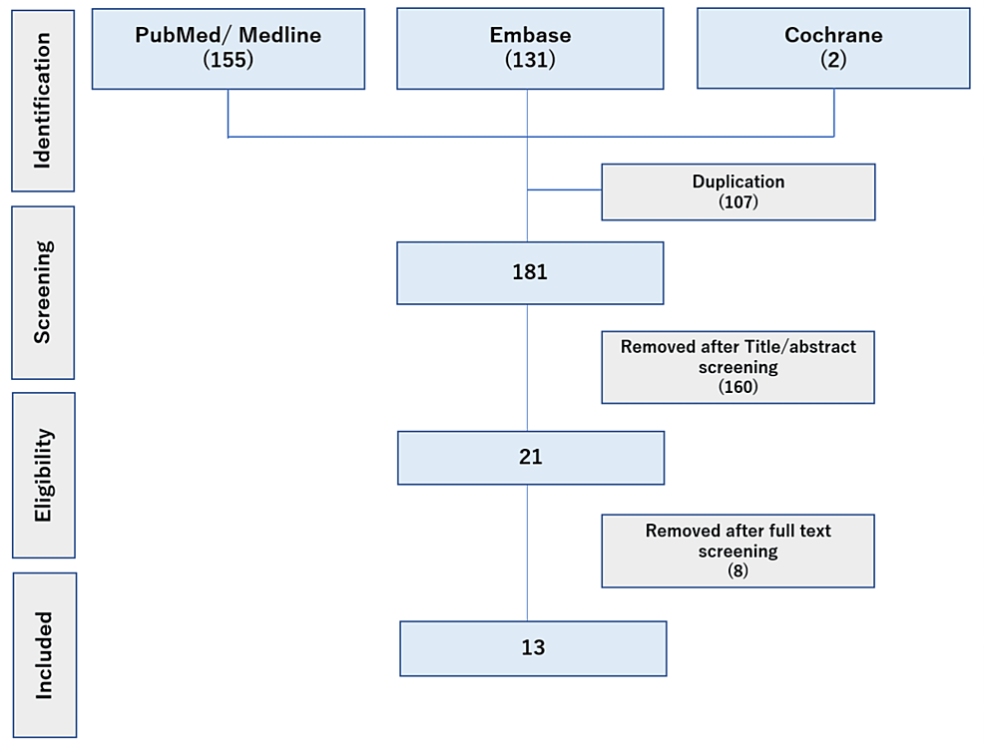
PRISMA flow of articles selection process PRISMA: Preferred Reporting Items for Systematic Reviews and Meta-Analyses

A total of 181 records were detected from our search strategy. The details of the screening process at each step are shown in Figure 1. In total, 13 studies met the eligibility criteria, and the country of origin of the studies included the United States, France, Italy, Poland, Belgium, Sweden, Germany, and the United Kingdom. Three were longitudinal studies, nine were cross-sectional in design, and one was a case study (detailed characteristics of each study are shown in Table 2). In line with the epidemiology of the LiS population, eight studies mainly include LiS patients with vascular etiology mostly due to stroke, two studies targeted LiS caused by ALS which is often a gradual process that takes several years, one study included patients with LiS (vascular or tumoral etiology) and ALS without LiS. Two studies had no data about the etiology of LiS. While the disease duration varied across studies, most examined stable LiS patients in which the disease period was over two years, except for one study [10]. The detailed characteristics of patients are shown in Table 3.

**Table 2:** Basic information of the 13 studies included in the scoping review ACSA: Anamnestic comparative self-assessment, SEIQoL-DW: Schedule for the evaluation of individual quality of life-direct weighting, ALS: Amyotrophic lateral sclerosis, ADI-12: ALS depression Inventory–12, SAHD: Schedule of attitudes toward hastened death, BDI-II: Beck depression inventory, TAS: Tront alexithymia scale, IPA-E: Impact on participation and autonomy, QoL: Quality of life, EQ-5D: Euro QOL 5 dimensions, STAI-Y: State trait anxiety inventory, HAM-A: Hamilton anxiety rating scale, HADS: Hospital anxiety and depression scale, MQoL: McGill QoL

ID	Author & year of publication	Design	Country	Objectives	Sample size (male)	Methods of psychological QOL	Religious yes/no	End-of-life issue
1	Kuzma-Kozakiewicz et al., 2019 [11]	Cross-sectional	Poland	Well-being and end-of-life preferences	19 (13)	ACSA, SEIQoL-DW, ADI-12, SAHD, Motor Neuron Disease Coping Scale	-	○
2	Rousseau et al., 2015 [12]	Longitudinal	France	The course of the QoL over 6 years and determine the potential contribution	67 (41) in 2007, 39 (24) in 2013	ACSA, questionnaire for psychological status	46/19	○
3	Rousseau et al., 2013 [13]	Cross-sectional	France	Compared QoL of LiS with healthy controls	No data	MQoL, SF-36, BDI-II,TAS	-	-
4	Snoeys et al., 2013 [14]	Cross-sectional	Belgium	Explore the situation of chronic LiS including QoL	8 (4)	SF-36, specific questions related to aspects relevant to changes due to LIS	-	○
5	Svernling et al., 2018 [15]	Cross-sectional	Sweden	Explore LiS in Sweden characteristics including QoL	10 (7)	RAND-36, IPA-E and EQ-5D	-	-
6	Linse et al., 2017 [16]	Cross-sectional	Germany	Assess QoL and psychological well-being of LiS	11 (6)	ADI-12, WHO-5, SEIQoL-DW	-	-
7	Bruno et al., 2011 [17]	Cross-sectional	France	Self-assessed QoL in chronic LiS	65 (43)	ACSA	40/17	○
8	Rousseau et al., 2011 [18]	Cross-sectional	France	Compare QoL of ALS and LiS with and without invasive ventilation	34 (22)	MQoL, SF-36, BDI-II, TAS, STAI-Y	-	-
9	Doble et al., 2003 [4]	Longitudinal	USA	Long-term outcome of patients with LiS	29 (19)	Original questionnaire for satisfaction with life and end of life	-	○
10	Nizzi et al., 2011 [19]	Cross-sectional	France	(A) global evaluation of identity, (B) body representation, (C) meaning in life	44 (30)	Original three-part questionnaire	-	-
11	Bernheim et al., 2019 [20]	Cross-sectional	Belgium	Effectiveness of ACSA as a QoL tool on various patients	2500 *diverse patients not only LIS	ACSA	-	-
12	Corallo et al., 2017 [10]	Longitudinal	Italy	The impact of the AAC on the QoL of LiS and caregivers	15 (9)	SF-36, HAM-A, BDI-II	-	-
13	Wilson et al., 2011 [21]	Case report	UK	Neuropsychological assessment of LiS	1 (0)	HADS, SF-36	-	-

**Table 3:** Characteristics of participants included in each study PEG: Percutaneous endoscopic gastrostomy, IV: Invasive ventilation, AAC tool: Alternative augmentative communication tool, ALS: Amyotrophic lateral sclerosis, LiS: Locked-in syndrome

ID	Author & year of publication	Mean age *Median	Etiology of LiS	Mean time from onset *Median time	Physical status PEG/IV	Caregiver/environment	Communication style: Eye-blink/eye-chat/etc.
1	Kuzma-Kozakiewicz et al., 2019 [11]	59	19: ALS	92 months	17: IV, 18: PEG	16: Partner, 2: Children 1: Professional	9: Eye-tracking, 6: Eye-blink 3: Combination 1: Residual verbal
2	Rousseau et al., 2015 [12]	47 in 2007, 51 in 2013	In 2007—51 Stroke, 8: Traumatic, 3: Others; In 2013—31: Stroke, 4: Traumatic, 3: Others	8 years in 2007, 14 years in 2013	In 2007—22: IV, 20: PEG; Patients with IV and PEG declined in 2013	In 2007—20: Institutional, 26: home; In 2013—6: Institutional, 27: Home	77%: Yes/No code, 58%: Computer communication device in 2007
3	Rousseau et al., 2013 [13]	No data	1: Trauma, Others: Vascular etiologies	No data	No data	No data	No data
4	Snoeys et al., 2013 [14]	41.1	7: Stroke, 1: Trauma	6 years 8 months	0: IV, 6: PEG	8: Home	1: verbal, 5: Partly verbal 4: Set of gestures, 7: Head & facial movement, 6: Eye codification
5	Svernling et al., 2018 [15]	49	7: Ischemic stroke, 3: Hemorrhagic	5.9 years	IV: No data, 2: PEG	2: Nursing home, 1: Apartment with society support, 4: Independent, 3: No data	1: Oral communication, Others: Letter boards/eye-tracking computer device or blinking
6	Linse et al., 2017 [16]	54.7	11: ALS	6.5 years (from ALS onset)	82%: IV, 82%: PEG	10: Home with 24-hour nursing care	11: Eye-tracking
7	Bruno et al., 2011 [17]	49	Most due to acute anterior pontine leison	8 years	No data	42: Home, 23: Institution	Only data about speech production— None: 45%, Words: 19%, Sentence: 36%
8	Rousseau et al., 2011 [18]	56.7	27: ALS, 7: LiS (6: vascular, 1:tumoral)	29 months	12: IV, 19: PEG	34: Hospital	No data
9	Doble et al., 2003 [4]	33.6	48%: Vascular, 34%: trauma, 10: hypotension, 2: Others	>11 years at study end	7: IV, 19: PEG at initiation, 2: IV, 6: PEG at 11 years after	8: Live with family, 3: Care facility, 1: State-run school, 1: Hospital with nursing	4: Computer use, 3:Letter board, 3: Facial movement, 2: Limb movement, 1: Vocalizations
10	Nizzi et al., 2011 [19]	53	No data	No data	No data	All at home	Eye-blink
11	Bernheim et al., 2019 [20]	no data	No data	No data	No data	No data	No data
12	Corallo et al., 2017 [10]	48.65	15: hemorrhage	1 month after onset at T0, 3 months after T0 at T1	No data	15: Hospital at T0, 8: Hospital, 7: Own home at T1	15: The AAC tool
13	Wilson et al., 2011 [21]	29	Basilar artery thrombosis	>2 years	1: PEG	1: Hospital	1: Letter board

Across the studies, 17 different instruments for QoL and psychological well-being assessment were utilized. Among them, 15 were established or validated tools with a standard score or comparable score based on the general population, and two were original questionnaires created for the studies [4,19]. The most common tool used was the SF-36 (RAND-36) [22], adopted by six studies. The anamnestic comparative self assessment (ACSA) [23] was used in four studies. Each of the following instruments was used in two studies: schedule for the evaluation of individual quality of life-direct weighting (SEIQoL-DW) [24], McGill QoL [25], Beck depression inventory (BDI-II) [26], and ALS depression inventory-12 items (ADI-12) [27]. Within 16 established instruments, three measured HRQoL (SF-36, World Health Organization well-being index (WHO-5) [28], EuroQol- 5 dimension (EQ-5D) [29], and three measured global QoL, (ACSA, SEIQoL-DW, McGill QoL). Nine instruments were used to measure the psychological status of depression, anxiety, autonomy, coping, and alexithymia. The last instrument was a questionnaire for assessing end-of-life issues (as seen above in Table 3). Overall, most studies showed reasonable results regarding psychological well-being and QoL across the different etiologies of LiS.

Findings From HRQoL Designed for General Population

Seven studies included an HRQoL assessment. The SF-36 (RAND-36) was used in six studies [8,13-15,21] and each WHO-5 and EQ-5D was used in one study, respectively [16]. Two studies with SF-36 [16] provided reasonable results, in which the LiS patients group showed a score nearly matched to the control or norm group. The other four studies with SF-36 did not set the control or reference group, however, two of them showed the score of the mental health domain at 75 and 90, respectively [15,7]. And one study provided a mean SF-36 score of 75.1 (in patients without invasive ventilation) and 74.6 (in patients with invasive ventilation) [18]. These scores correspond to the general population in a previous report from Wales [30]. One study with WHO-5 also presented reasonable results in LiS patients with a mean score of 63.6 (the reference score of WHO-5 is 60.0). The EQ-5D in one study showed negative values in which three of four participants reported values of less than 0 (a score under 0 in EQ-5D is described as indicating a condition worse than death). However, the authors interpreted this result as physical functioning having a significant impact on EQ-5D results.

Findings From Global QoL Designed for Intractable Diseases (Subjective QoL)

Seven studies utilized the global QoL for intractable diseases [11-13,16-18,20] and showed reasonable results with the exception of one study [20]. Among the four studies using the ACSA, three showed reasonable outcomes presenting mean values of greater than 0 or a majority of participants with a value of greater than 0 [11,12,17]. One study recruited a diverse series of patients including LiS and reported LiS as a major subgroup that reported poor outcomes based on the ACSA [20]. Considering that the ACSA incorporates features of a highly individualized biographical scale, ACSA might not be a suitable tool for relative comparisons.

Findings From Questionnaires for Psychological Status

Among nine studies that included this type of assessment, seven administered assessment tools for depression, including ADI-12, BDI, the hospital anxiety and depression scale (HADS) [31], and an original questionnaire. Among three studies using the BDI, two indicated borderline clinical depression with scores of 17 or greater or moderate depression in LiS patients [10,18], and one study reported more frequent depressive symptoms in the LiS group than in healthy controls [20]. Whereas a study using HADS showed almost no symptoms of depression with a score of 1. In two studies, ADI-12, which was developed for ALS patients, also showed a normal state in LiS patients with mean scores of 25 [11], and 19.7 [16]. One study asked about the presence of depression and showed that 13% answered being depressed [28].

Regarding anxiety, the state-trait anxiety inventory (STAI-Y) [32], Hamilton anxiety rating scale (HAM-A) [33], and HADS were used in one study, and two used original questionnaires. The STAI-Y presented clinically significant anxiety symptoms in LiS patients with each of the basal (trait) and reactive (state) scores showing greater than 50 [18]. The HAM-A showed mild-moderate anxiety in LiS patients with a mean score of 21 at baseline, but after three months indicated normal with a mean score of 16 [10]. The HADS showed a normal status with a score of 5 [21]. One study asked about the anxiety status (none, moderate, extreme) and showed 54% of patients with moderate anxiety and 13% with extreme [17]. Another study inquired about the presence of anxiety/depression and reported 55% of patients with anxiety and/or mood disorders at baseline [12].

One study examined participation/autonomy by the impact on participation and autonomy (IPA-E I) [34]. Results showed a reasonable score compared to the reference data [15]. Coping status was measured by the motor neuron disease coping scale [35] in one study and showed that information-seeking increased with time since diagnosis only [11]. Alexithymia was assessed by the Toronto alexithymia scale (TAS) [36] in one, and it showed possible alexithymia with scores of 56.9 and 60.3 [18].

Perception of Psychological Well-Being Between Patients and Caregiver/Next of Kin

Two studies showed similar results in which caregivers or next of kin (NOK) tended to rate the patients' QoL lower and overestimated patients' depression, but the differences were not significant [16,17]. These results were in line with previous reports for other fatal disorders, cancer patients, and caregivers [37]. Researchers suggested that the differences may be due to inconsistencies between patients and NOK in terms of important features of life that are of value, and it appeared to reflect the successful adaption (adaptation) to the disease by the patients but not by the NOK [16].

End-of-Life Issues

Five studies assessed end-of-life issues [4,11,12,14,17]. One study used the schedule of attitudes toward hastened death (SAHD) [38], and participants presented a middle-level wish for hastened death with a mean score of 4.5 [11]. Across the remaining four studies, participants were asked about suicidal thoughts and euthanasia and studies reported a range of 27% to 68% having these types of thoughts (Table 4). Patients in these five studies all had stable LiS with over six years of disease duration.

**Table 4:** Studies assessing end-of-life issues in participants SAHD: Schedule of attitudes toward hastened death

ID	Author & year of publication	Sample size (male)	End-of-life issue
1	Kuzma-Kozakiewicz et al., 2019 [11]	19 (13)	Patients presented with a median wish for hastened death of 4.5 of the SAHD score
2	Rousseau et al., 2015 [12]	67 (41) in 2007, 39 (24) in 2013	27% had suicidal thoughts and 2 reported a wish for euthanasia in 2007. No one wished for euthanasia and 3 reported new suicidal ideas in 2013.
4	Snoeys et al., 2013 [14]	8 (4)	Suicidal thought—5: Never, 1: Did in the past, 1: Sometimes, 1: Often; Euthanasia—4: Never, 2: In the past, 2: Currently
7	Bruno et al., 2011 [17]	65 (43)	Suicidal thought—68%: Never, 24%: Occasionally, 8%: Often; Euthanasia—47%: Never, 53%: Envisaged
9	Doble et al., 2003 [4]	29 (19)	Euthanasia/suicidal thought from reported resource—7: Never, 6: considered in the past but not currently, 1: Wish to die

Influential Factors on Psychological Well-Being

Seven studies examined factors associated with QoL or well-being. Disease duration was a factor assessed most commonly. Among four studies, half found longer duration to be a factor positively affecting QoL [16,17], while the other two did not find a relation [11,12]. Regarding the influence of communication methods, AAC tools and recovery of speech production showed positive effects [10,17], whereas the use of the yes/no code showed a negative impact on QoL [29]. Thus, the communication modalities with high flexibility appeared to be positively associated with patients’ well-being. Among the studies that performed an assessment, most found that physical function had no relation to QoL, rather one study showed that disease severity was positively correlated with QoL. In addition, Kuzna et al. reported that LiS patients in ALS with no residual physical function (ALSFRS-R=0) showed positive QoL and no clinically significant depression [11,15]. Experiencing anxiety and suicidal thoughts were found to be negatively associated with QoL [12,17].

Global QoL and HRQoL results did not show marked patterns regarding the country of the study. However, for the depression scales, there appeared to be a tendency for negative results in studies from southwestern Europe, France, and Italy [10,13,18], and reasonable or normal results in studies from central Europe, Poland, and Germany [11,16]. However, different depression scales were used and the results may be due to the type of depression instruments rather than geographic character. Studies with reasonable results used ADI-12 [16,18], which is a depression scale developed for ALS and patients with limited physical function, whereas studies with negative results for depression used BDI [8,14,28]. The BDI has the possibility to overvalue depression in patients with physical function loss because the scores of BDI are susceptible to physical disability [39].

Discussion

The present study is, to our knowledge, the first review to synthesize the evidence regarding the psychological well-being of patients with LiS. Across the 13 eligible articles, we observed that patients with LiS had reasonable or similar psychological well-being as the standard regardless of etiology, socio-demography, and physical disability level. Caregivers and healthcare professionals seem to rate the psychological QoL of LiS patients lower than patients themselves. This tendency is consistent with other fatal disorders [37,40]. Linse et al. explained that this may reflect the successful adaptation of the patients to the disease and that a “response shift” may have occurred in patients with LiS [16]. Psychological adaptation has also been described by others [11,12,17,19] and is a well-known phenomenon in diseases such as ALS or advanced-stage cancer [37,40-42].

Sprangers et al. defined the response shift as involving: 1) recalibration for a new scale in measuring one’s state of quality, 2) reprioritization that represents a change in the priority of values influencing one’s own QoL, and 3) reconceptualization consisting of a reconstruction of one’s concept; for example, patients place greater weight on inner and spiritual value after serious illness [43]. We can consider these shifts as an important process for adaptation among LiS patients influencing the QoL in a positive manner. Global QoL (subjective QoL) may be influenced more sharply by the response shift compared to HRQOL, such as EQ-5D.

Considering the importance of adaptation for the well-being of LiS patients, it seems reasonable to observe that disease duration was associated with successful psychological outcomes. Studies showed a positive correlation between disease duration and psychological well-being. Among the studies in this review, there were three longitudinal studies with a follow-up period of six years, 11 years, and three months [4,10,12]. The study with the six-year follow-up showed no significant difference in ACSA score within the period, however, the prevalence of anxiety and mood disorder declined from 55% to 31%. The study with the three-month follow-up showed meaningful improvement in SF-36 score, depression, and anxiety, while we need to consider that acquisition of communication ability with the AAC tool in this study period may have impacted the QoL status. The study with an 11-year follow-up did not provide a comparison of well-being across the observation period. Considering the duration from the time of onset, all seven studies including patients with a disease duration of greater than five years showed reasonable results or similar scores to the standard population regarding QoL or psychological well-being [4,11,12,14-17]. In contrast, two studies with a disease duration of less than three years showed negative aspects among the psychological domains [10,18]. In one case study, the patient with a two-year disease duration reported a reasonable QoL and reported better general health than a year ago [21]. 

As another description of the effect of adaptation, Kuzma et al. reported that patients with good psychosocial adaptation were also the ones who were well-informed [11]. Interestingly, well-informed and transparent information was also identified as unfulfilled needs of LiS patients in other studies [14,15]. Transparent information includes the precise pathophysiology of LiS, its prognosis, and the latest technologies including advanced communication devices, social support systems, and proper care. There is a sentiment that this information should be given to the patients with respect, and caregivers (and the public) would benefit by obtaining an understanding similar to that of the patients [15].

Given that disease duration and being well-informed is associated with the adaptation and well-being of patients, end-of-life decisions in the early phase of LiS should be provided discretely. Regarding the end-of-life issue and thoughts of suicide and euthanasia in the present review, LiS patients presented a middle-level wish for hastened death, however, these ideas tended to have decreased over time. Bruno et al. also suggested that a moratorium should be proposed for patients with suicidal thoughts in the acute setting [17]. At the same time, the patient’s perception of being a burden to caregivers was identified as a determinant of wishing for a hastened death, as well as for depression, anxiety, and poor QoL [44]. Thus, when we consider the end-of-life issue for LiS patients, it may be meaningful to also consider issues of caregiver burden, and not only support focused on the patients. Financial aid and social support, such as respite care (short-term assistance serving rest and relief for caregivers) may be a vitally important resource for both patients and caregivers [45].

Communication methods with high flexibility are also important to improve QoL in patients with LiS. Studies indicate that the AAC tool and eye-tracking computer system (ETCS) could help improve the QoL of patients [10,16]. However, when the possibility is available, recovery of speech products is a better way to communicate naturally and may be a positive factor for the well-being of patients. Most studies of LiS patients with vascular etiology in this review noted the importance of multidisciplinary rehabilitation because it might improve the prognosis and can make LiS patients regain voluntary head control, finger movement, and sometimes partial speech production. A rehabilitation plan and strategy for LiS should be developed in an individualized fashion considering the disease phase due to the variation in LiS pathophysiology.

For the clinical setting, transparent information should be provided to both LiS patients and caregivers, and healthcare professionals and caregivers should also understand the actual psychological QoL of patients without pre-conceptions. A sufficient moratorium period should be secured for patients to support decision-making. Previous studies have shown that low health literacy is associated with low QoL [46]. The selection of an appropriate QoL instrument is also important. The HRQoL and general tools for assessing depression could underestimate the QoL state and overestimate depression in patients with LiS [39]. There is a need to develop suitable instruments for assessing psychological well-being specific to LiS patients to understand their mental health accurately. 

For policy setting, it is necessary to provide broad social support mechanisms including financial, medical, communication devices, and a system of social support to both patients and caregivers to help secure their dignity, autonomy, and wellness. Hofman et al. reported on the connections between the improvement of social support and an increase in the QoL of lung cancer patients, another fatal disease [47].

There are limitations to acknowledge regarding this scoping review. Some included studies noted that patients who could not participate in the study might have had poor QoL. Excluded subjects from the studies may be those with limited interactions with society and severe physical and mental situations. Thus, this review may not be appropriately capturing the circumstances of these subsets of patients. From the present review, we did not observe different tendencies for psychological QoL across LiS of different etiologies. However, the time course from underlying disease onset to complete LiS varies depending on etiology. For example, LiS caused by ALS develops gradually, while LiS with a vascular etiology is sudden. Thus, the coping and adaptation processes of patients may be different. It can be adapted to the rehabilitation strategy. The rehabilitation for sudden onset LiS sometimes aims to regain motor function even if it is only limited recovery, while rehabilitation for LiS in ALS or other fatal neurodegenerative diseases tends to maintain residual functionality for longer periods. Thus, further research for developing the care and coping strategy for LiS by etiology and type of LiS will be a significant contribution.

## Conclusions

In reviewing the 13 studies that met eligible criteria, we found that patients with LiS generally had reasonable or similar psychological well-being to the standard population. Caregivers' perceptions and the patients' assessed QoL were observed to be different in some studies. Response shift and adaptation to disease by patients (not by caregivers) are potential reasons for this gap. Disease duration and being well-informed with transparency may influence this process. Given these observations, it seems essential to provide a sufficient moratorium period and appropriate information including social support and care options for patients to aid the decision-making process.

## References

[1] (2022). Locked In Syndrome - National Organization for Rare Disorders. https://rarediseases.org/rare-diseases/locked-in-syndrome/?filter=Causes.

[2] Laureys S, Pellas F, Van Eeckhout P (2005). The locked-in syndrome: what is it like to be conscious but paralyzed and voiceless?. Prog Brain Res.

[3] Bauer G, Gerstenbrand F, Rumpl E (1979). Varieties of the locked-in syndrome. J Neurol.

[4] Doble JE, Haig AJ, Anderson C (2003). Impairment, activity, participation, life satisfaction, and survival in persons with locked-in syndrome for over a decade: follow-up on a previously reported cohort. J Head Trauma Rehabil.

[5] Kohnen RF, Lavrijsen JC, Bor JH (2013). The prevalence and characteristics of patients with classic locked-in syndrome in Dutch nursing homes. J Neurol.

[6] (2022). NORD -National Organization for Rare Disorders: Locked-In Syndrome. https://rarediseases.org/rare-diseases/locked-in-syndrome/?filter=Signs+%26+Symptoms.

[7] Halan T, Ortiz JF, Reddy D (2021). Locked-in syndrome: a systematic review of long-term management and prognosis. Cureus.

[8] Arksey H, O'Malley L (2005). Scoping studies: towards a methodological framework. Int J Soc Res Methodol.

[9] Levac D, Colquhoun H, O'Brien KK (2010). Scoping studies: advancing the methodology. Implement Sci.

[10] Corallo F, Bonanno L, Lo Buono V (2017). Augmentative and alternative communication effects on quality of life in patients with locked-in syndrome and their caregivers. J Stroke Cerebrovasc Dis.

[11] Kuzma-Kozakiewicz M, Andersen PM, Ciecwierska K (2019). An observational study on quality of life and preferences to sustain life in locked-in state. Neurology.

[12] Rousseau MC, Baumstarck K, Alessandrini M (2015). Quality of life in patients with locked-in syndrome: evolution over a 6-year period. Orphanet J Rare Dis.

[13] Rousseau MC, Pietra S, Nadji M (2013). Evaluation of quality of life in complete locked-in syndrome patients. J Palliat Med.

[14] Snoeys L, Vanhoof G and Manders E (2013). Living with locked-in syndrome: an explorative study on health care situation, communication and quality of life. Disabil Rehabil.

[15] Svernling K, Tornbom M, Nordin A (2019). Locked-in syndrome in Sweden, an explorative study of persons who underwent rehabilitation: a cohort study. BMJ Open.

[16] Linse K, Ruger W, Joos M (2017). Eye-tracking-based assessment suggests preserved well-being in locked-in patients. Ann Neurol.

[17] Bruno MA, Bernheim JL, Ledoux D (2011). A survey on self-assessed well-being in a cohort of chronic locked-in syndrome patients: happy majority, miserable minority. BMJ Open.

[18] Rousseau MC, Pietra S, Blaya J (2011). Quality of life of ALS and LIS patients with and without invasive mechanical ventilation. J Neurol.

[19] Nizzi MC, Demertzi A, Gosseries O (2012). From armchair to wheelchair: how patients with a locked-in syndrome integrate bodily changes in experienced identity. Conscious Cogn.

[20] Bernheim JL (2019). Personalised felicitometrics. ACSA, a self-anchoring uniscale based on life experience, may circumvent several biases. 26th Annual Conference of the International Society for Quality of Life. Qual Life Res.

[21] Wilson BA, Hinchcliffe A, Okines T (2011). A case study of locked-in-syndrome: psychological and personal perspectives. Brain Inj.

[22] McHorney CA, Ware JE Jr, Lu JF, Sherbourne CD (1994). The MOS 36-item Short-Form Health Survey (SF-36): III. Tests of data quality, scaling assumptions, and reliability across diverse patient groups. Med Care.

[23] Bernheim JL, Buyse M (1983). The amnestic comparative self-assessment for measuring the subjective quality of life in cancer patients 1993. J Psychosoc Oncol.

[24] Hickey AM, Bury G, O'Boyle CA (1996). A new short form individual quality of life measure (SEIQoL-DW): application in a cohort of individuals with HIV/AIDS. BMJ.

[25] Cohen SR, Russell LB, Leis A (2019). More comprehensively measuring quality of life in life-threatening illness: the McGill Quality of Life Questionnaire - expanded.. BMC Palliat Care.

[26] Beck Beck, A.T. A.T., Steer Steer, R.A. R.A., & Brown, G G (2022). Beck Depression Inventory-Second Edition | The National Child Traumatic Stress Network. https://www.nctsn.org/measures/beck-depression-inventory-second-edition.

[27] Hammer EM, Hacker S, Hautzinger M (2008). Validity of the ALS-Depression-Inventory (ADI-12)—a new screening instrument for depressive disorders in patients with amyotrophic lateral sclerosis. J Affect Disord.

[28] Topp CW, Ostergaard SD, Sondergaard S (2015). The WHO-5 well-being index: a systematic review of the literature. Psychother Psychosom.

[29] EuroQol Research Foundation (2022). EQ-5D. https://euroqol.org.

[30] Burholt V, Nash P (2011). Short Form 36 (SF-36) health survey questionnaire: normative data for Wales. J Public Health (Oxf).

[31] Zigmond AS, Snaith RP (1983). The hospital anxiety and depression scale. Acta Psychiatr Scand.

[32] Spielberger CD, Gorsuch RL, Lushene R, Vagg PR (1983). Manual for the State-Trait Anxiety Inventory (Form Y1-Y2). Manual for the State-Trait Anxiety Inventory (Form Y1-Y2).

[33] Hamilton M (1959). The assessment of anxiety states by rating. Br J Med Psychol.

[34] Kersten P, Cardol M, George S, Ward C, Sibley A, White B (2007). Validity of the impact on participation and autonomy questionnaire: a comparison between two countries. Disabil Rehabil.

[35] Lee JN, Rigby SA, Burchardt F, Thornton EW, Dougan C, Young CA (2001). Quality of life issues in motor neurone disease: the development and validation of a coping strategies questionnaire, the MND Coping Scale. J Neurol Sci.

[36] Taylor GJ, Ryan D, Bagby RM (1985). Toward the development of a new self-report alexithymia scale. Psychother Psychosom.

[37] Kassir ZM, Li J, Harrison C (2021). Disparity of perception of quality of life between head and neck cancer patients and caregivers. BMC Cancer.

[38] Galushko M, Strupp J, Walisko-Waniek J (2015). Validation of the German version of the schedule of attitudes toward hastened death (SAHD-D) with patients in palliative care. Palliat Support Care.

[39] Pagnini F, Manzoni GM, Tagliaferri A (2015). Depression and disease progression in amyotrophic lateral sclerosis: a comprehensive meta-regression analysis. J Health Psychol.

[40] Lule D, Ehlich B, Lang D (2013). Quality of life in fatal disease: the flawed judgement of the social environment. J Neurol.

[41] Lule D, Hacker S, Ludolph A (2008). Depression and quality of life in patients with amyotrophic lateral sclerosis. Dtsch Arztebl Int.

[42] Neudert C, Wasner M and Borasio GD (2004). Individual quality of life is not correlated with health-related quality of life or physical function in patients with amyotrophic lateral sclerosis. J Palliat Med.

[43] Sprangers MA and Schwartz CE (1999). Integrating response shift into health-related quality of life research: a theoretical model. Soc Sci Med.

[44] Stutzki R, Weber M, Reiter-Theil S (2014). Attitudes towards hastened death in ALS: a prospective study of patients and family caregivers. Amyotroph Lateral Scler Front Degener.

[45] Wu JM, Tam MT, Buch K (2022). The impact of respite care from the perspectives and experiences of people with amyotrophic lateral sclerosis and their care partners: a qualitative study. BMC Palliat Care.

[46] Panagioti M, Skevington SM, Hann M (2018). Effect of health literacy on the quality of life of older patients with long-term conditions: a large cohort study in UK general practice. Qual Life Res.

[47] Hofman A, Zajdel N, Klekowski J (2021). Improving social support to increase QoL in lung cancer patients. Cancer Manag Res.

